# Supplementary material for: Multi‐dataset identification of innovative feature genes and molecular mechanisms in keratoconus
Source: J Cell Mol Med. 2024 Sep 19;28(18):e70079. doi: 10.1111/jcmm.70079 (PMC11412914; doi:10.1111/jcmm.70079)
Supplement: Supplementary file 2 — Material S2. [file JCMM-28-e70079-s002.zip › 7.GSEA analysis/6.SNORA74B/KEGG_PURINE_METABOLISM.html]

Details for gene set KEGG\_PURINE\_METABOLISM[GSEA]

|  || Dataset | SNORA74B.group.cls#h\_versus\_l |
| Phenotype | group.cls#h\_versus\_l |
| Upregulated in class | l |
| GeneSet | KEGG\_PURINE\_METABOLISM |
| Enrichment Score (ES) | -0.33377388 |
| Normalized Enrichment Score (NES) | -1.4709858 |
| Nominal p-value | 0.055009823 |
| FDR q-value | 0.32829398 |
| FWER p-Value | 0.823 |
Table: GSEA Results Summary

  

Fig 1: Enrichment plot: KEGG\_PURINE\_METABOLISM      
 Profile of the Running ES Score & Positions of GeneSet Members on the Rank Ordered List

  

| SYMBOL | TITLE | RANK IN GENE LIST | RANK METRIC SCORE | RUNNING ES | CORE ENRICHMENT || 1 | PDE7A | na | 488 | 0.607 | 0.0144 | No |
| 2 | NT5E | na | 762 | 0.530 | 0.0314 | No |
| 3 | ENTPD8 | na | 771 | 0.528 | 0.0560 | No |
| 4 | PDE3B | na | 2263 | 0.350 | 0.0293 | No |
| 5 | ADCY2 | na | 2866 | 0.309 | 0.0264 | No |
| 6 | NT5C1B | na | 3039 | 0.298 | 0.0354 | No |
| 7 | NT5C2 | na | 5523 | 0.208 | -0.0266 | No |
| 8 | ADCY5 | na | 6025 | 0.192 | -0.0321 | No |
| 9 | ADCY7 | na | 6070 | 0.190 | -0.0244 | No |
| 10 | ENTPD2 | na | 6213 | 0.185 | -0.0198 | No |
| 11 | GUCY1A2 | na | 6240 | 0.185 | -0.0119 | No |
| 12 | PDE4D | na | 6530 | 0.176 | -0.0120 | No |
| 13 | ADCY4 | na | 7191 | 0.157 | -0.0237 | No |
| 14 | PDE6H | na | 7453 | 0.150 | -0.0242 | No |
| 15 | NPR2 | na | 7602 | 0.146 | -0.0216 | No |
| 16 | AK5 | na | 7608 | 0.146 | -0.0149 | No |
| 17 | POLR1A | na | 7696 | 0.143 | -0.0107 | No |
| 18 | AK4 | na | 7873 | 0.139 | -0.0093 | No |
| 19 | POLE | na | 8968 | 0.114 | -0.0355 | No |
| 20 | ENTPD6 | na | 9092 | 0.111 | -0.0339 | No |
| 21 | PDE4C | na | 9143 | 0.110 | -0.0301 | No |
| 22 | PAPSS1 | na | 10725 | 0.079 | -0.0721 | No |
| 23 | ADCY9 | na | 10911 | 0.076 | -0.0739 | No |
| 24 | ADCY1 | na | 11165 | 0.072 | -0.0778 | No |
| 25 | GDA | na | 11871 | 0.062 | -0.0953 | No |
| 26 | NUDT2 | na | 11912 | 0.061 | -0.0935 | No |
| 27 | ALLC | na | 12023 | 0.060 | -0.0939 | No |
| 28 | POLR2H | na | 12060 | 0.059 | -0.0922 | No |
| 29 | ENPP3 | na | 12163 | 0.058 | -0.0924 | No |
| 30 | ITPA | na | 12224 | 0.057 | -0.0915 | No |
| 31 | POLR2J2 | na | 12274 | 0.056 | -0.0903 | No |
| 32 | IMPDH1 | na | 12385 | 0.055 | -0.0909 | No |
| 33 | NT5C | na | 12413 | 0.054 | -0.0891 | No |
| 34 | PDE6C | na | 12415 | 0.054 | -0.0866 | No |
| 35 | FHIT | na | 12460 | 0.054 | -0.0853 | No |
| 36 | ADCY6 | na | 12561 | 0.052 | -0.0857 | No |
| 37 | NPR1 | na | 12582 | 0.052 | -0.0839 | No |
| 38 | POLR3H | na | 12698 | 0.051 | -0.0848 | No |
| 39 | POLR3B | na | 14056 | 0.035 | -0.1224 | No |
| 40 | POLD4 | na | 14153 | 0.034 | -0.1236 | No |
| 41 | PDE6A | na | 14310 | 0.032 | -0.1266 | No |
| 42 | POLR2C | na | 14386 | 0.031 | -0.1273 | No |
| 43 | XDH | na | 14497 | 0.030 | -0.1290 | No |
| 44 | GUCY2F | na | 14628 | 0.029 | -0.1315 | No |
| 45 | POLD1 | na | 14676 | 0.028 | -0.1315 | No |
| 46 | ENTPD4 | na | 14753 | 0.027 | -0.1324 | No |
| 47 | POLR2A | na | 15608 | 0.018 | -0.1563 | No |
| 48 | PPAT | na | 15898 | 0.016 | -0.1639 | No |
| 49 | POLR2J3 | na | 16190 | 0.013 | -0.1717 | No |
| 50 | PRPS1 | na | 16331 | 0.011 | -0.1752 | No |
| 51 | GUCY2D | na | 16346 | 0.011 | -0.1751 | No |
| 52 | NME3 | na | 16386 | 0.011 | -0.1757 | No |
| 53 | PDE9A | na | 16805 | 0.006 | -0.1875 | No |
| 54 | POLR3A | na | 16982 | 0.005 | -0.1924 | No |
| 55 | POLR2I | na | 17022 | 0.005 | -0.1933 | No |
| 56 | POLR2J | na | 17188 | 0.003 | -0.1979 | No |
| 57 | GART | na | 17310 | 0.001 | -0.2014 | No |
| 58 | POLR1C | na | 17862 | -0.004 | -0.2171 | No |
| 59 | AK1 | na | 18029 | -0.006 | -0.2216 | No |
| 60 | APRT | na | 18165 | -0.008 | -0.2251 | No |
| 61 | ENTPD5 | na | 18344 | -0.010 | -0.2298 | No |
| 62 | AK2 | na | 18404 | -0.011 | -0.2310 | No |
| 63 | ADCY8 | na | 18678 | -0.014 | -0.2383 | No |
| 64 | NME5 | na | 18723 | -0.014 | -0.2389 | No |
| 65 | PDE10A | na | 18766 | -0.014 | -0.2394 | No |
| 66 | ADSL | na | 19022 | -0.017 | -0.2460 | No |
| 67 | GMPR2 | na | 19161 | -0.019 | -0.2491 | No |
| 68 | PNP | na | 19210 | -0.020 | -0.2495 | No |
| 69 | POLR2G | na | 19301 | -0.021 | -0.2511 | No |
| 70 | POLE4 | na | 19561 | -0.024 | -0.2575 | No |
| 71 | POLR2L | na | 19908 | -0.027 | -0.2662 | No |
| 72 | NME1 | na | 20060 | -0.029 | -0.2692 | No |
| 73 | POLR2E | na | 20158 | -0.030 | -0.2706 | No |
| 74 | AMPD3 | na | 20223 | -0.031 | -0.2710 | No |
| 75 | POLR2D | na | 20657 | -0.036 | -0.2819 | No |
| 76 | NUDT9 | na | 20840 | -0.038 | -0.2854 | No |
| 77 | NME2 | na | 20850 | -0.038 | -0.2838 | No |
| 78 | POLR1B | na | 21108 | -0.041 | -0.2893 | No |
| 79 | NUDT5 | na | 21125 | -0.041 | -0.2878 | No |
| 80 | ATIC | na | 21407 | -0.045 | -0.2938 | No |
| 81 | NT5C3A | na | 21461 | -0.045 | -0.2932 | No |
| 82 | DGUOK | na | 21497 | -0.046 | -0.2921 | No |
| 83 | NME6 | na | 21567 | -0.047 | -0.2919 | No |
| 84 | POLR2F | na | 21583 | -0.047 | -0.2901 | No |
| 85 | PDE6G | na | 21685 | -0.048 | -0.2908 | No |
| 86 | GUCY2C | na | 21778 | -0.049 | -0.2912 | No |
| 87 | PNPT1 | na | 21812 | -0.049 | -0.2898 | No |
| 88 | POLR2B | na | 21915 | -0.051 | -0.2904 | No |
| 89 | POLR3K | na | 22054 | -0.052 | -0.2919 | No |
| 90 | POLR1D | na | 22190 | -0.054 | -0.2933 | No |
| 91 | POLE3 | na | 22272 | -0.055 | -0.2930 | No |
| 92 | ADA | na | 22368 | -0.056 | -0.2931 | No |
| 93 | POLD2 | na | 22465 | -0.058 | -0.2932 | No |
| 94 | POLR3GL | na | 22632 | -0.060 | -0.2952 | No |
| 95 | GUK1 | na | 22744 | -0.061 | -0.2955 | No |
| 96 | GMPS | na | 22932 | -0.064 | -0.2979 | No |
| 97 | CANT1 | na | 22983 | -0.065 | -0.2963 | No |
| 98 | ENTPD3 | na | 23141 | -0.067 | -0.2976 | No |
| 99 | IMPDH2 | na | 23330 | -0.070 | -0.2998 | No |
| 100 | ADK | na | 23370 | -0.071 | -0.2976 | No |
| 101 | POLA1 | na | 23506 | -0.073 | -0.2980 | No |
| 102 | POLD3 | na | 23900 | -0.079 | -0.3057 | No |
| 103 | PRIM2 | na | 24056 | -0.082 | -0.3063 | No |
| 104 | RRM1 | na | 24063 | -0.082 | -0.3027 | No |
| 105 | PKM | na | 24276 | -0.085 | -0.3048 | No |
| 106 | PDE8A | na | 24384 | -0.087 | -0.3038 | No |
| 107 | POLR2K | na | 24648 | -0.092 | -0.3071 | No |
| 108 | PKLR | na | 24696 | -0.093 | -0.3041 | No |
| 109 | POLR3G | na | 25043 | -0.099 | -0.3094 | No |
| 110 | POLR1E | na | 25321 | -0.104 | -0.3125 | No |
| 111 | PDE6B | na | 25840 | -0.113 | -0.3222 | No |
| 112 | POLR3D | na | 25844 | -0.113 | -0.3169 | No |
| 113 | PDE2A | na | 25993 | -0.117 | -0.3157 | No |
| 114 | HPRT1 | na | 26087 | -0.118 | -0.3128 | No |
| 115 | PDE4A | na | 26569 | -0.129 | -0.3207 | No |
| 116 | ADCY3 | na | 26777 | -0.134 | -0.3203 | No |
| 117 | PAICS | na | 27101 | -0.142 | -0.3230 | No |
| 118 | PDE3A | na | 27126 | -0.142 | -0.3171 | No |
| 119 | PDE6D | na | 27702 | -0.157 | -0.3263 | Yes |
| 120 | NT5C1A | na | 27740 | -0.158 | -0.3200 | Yes |
| 121 | DCK | na | 28219 | -0.171 | -0.3257 | Yes |
| 122 | AMPD1 | na | 28270 | -0.172 | -0.3191 | Yes |
| 123 | ADPRM | na | 28489 | -0.179 | -0.3170 | Yes |
| 124 | POLR3F | na | 28601 | -0.182 | -0.3116 | Yes |
| 125 | PRPS2 | na | 28730 | -0.187 | -0.3066 | Yes |
| 126 | RRM2B | na | 28962 | -0.194 | -0.3041 | Yes |
| 127 | AK7 | na | 28973 | -0.194 | -0.2953 | Yes |
| 128 | ENTPD1 | na | 29007 | -0.195 | -0.2871 | Yes |
| 129 | PDE1C | na | 29035 | -0.196 | -0.2786 | Yes |
| 130 | NT5M | na | 29238 | -0.202 | -0.2750 | Yes |
| 131 | POLA2 | na | 29251 | -0.202 | -0.2658 | Yes |
| 132 | PRIM1 | na | 29730 | -0.216 | -0.2695 | Yes |
| 133 | NME7 | na | 29880 | -0.221 | -0.2634 | Yes |
| 134 | ADCY10 | na | 31288 | -0.283 | -0.2908 | Yes |
| 135 | PRPS1L1 | na | 31594 | -0.300 | -0.2855 | Yes |
| 136 | POLE2 | na | 31670 | -0.305 | -0.2734 | Yes |
| 137 | PDE7B | na | 31870 | -0.316 | -0.2643 | Yes |
| 138 | PFAS | na | 31889 | -0.317 | -0.2499 | Yes |
| 139 | NME4 | na | 32032 | -0.324 | -0.2388 | Yes |
| 140 | PDE11A | na | 32842 | -0.380 | -0.2443 | Yes |
| 141 | AMPD2 | na | 33036 | -0.397 | -0.2312 | Yes |
| 142 | ENPP1 | na | 33524 | -0.452 | -0.2241 | Yes |
| 143 | RRM2 | na | 33643 | -0.470 | -0.2054 | Yes |
| 144 | PAPSS2 | na | 33663 | -0.471 | -0.1838 | Yes |
| 145 | URAD | na | 33738 | -0.484 | -0.1632 | Yes |
| 146 | PDE5A | na | 33748 | -0.485 | -0.1407 | Yes |
| 147 | PDE1B | na | 33763 | -0.487 | -0.1182 | Yes |
| 148 | PDE4B | na | 34444 | -0.671 | -0.1063 | Yes |
| 149 | PDE1A | na | 34593 | -0.782 | -0.0738 | Yes |
| 150 | GMPR | na | 34608 | -0.804 | -0.0364 | Yes |
| 151 | PDE8B | na | 34634 | -0.848 | 0.0027 | Yes |
Table: GSEA details [plain text format]

  

Fig 2: KEGG\_PURINE\_METABOLISM      
 Blue-Pink O' Gram in the Space of the Analyzed GeneSet

  

Fig 3: KEGG\_PURINE\_METABOLISM: Random ES distribution      
 Gene set null distribution of ES for **KEGG\_PURINE\_METABOLISM**
